# Seasonal collection of in situ optical and thermal images dataset and meteorological measurements over an Indian semi-arid rice crop

**DOI:** 10.1016/j.dib.2026.112646

**Published:** 2026-03-02

**Authors:** Chandrika Pinnepalli, Jean-Louis Roujean, Marc Oliver-Soulayrol, Rahul Nigam, Mark Irvine, Ayan Das, Rucha Dave, Bimal K. Bhattacharya

**Affiliations:** a Université de Toulouse, CNES/IRD/CNRS/INRAe, CESBIO, Toulouse, 31400, France; b HSM, Univ. Montpellier, CNRS, IMT, IRD, Montpellier, 34095, France; c Space Applications Centre, ISRO, Ahmedabad, 380015, India; d INRAE, 71 Av. Edouard Bourlaux, 33140 Villenave-d'Ornon, France; e Anand Agricultural University, GXPF+9W5, Anand, Gujarat, 388110, India

**Keywords:** Thermal infrared, Anisotropy, Image processing, Ancillary measurements, Rice canopy, Meteorological Data

## Abstract

This article describes a multi-sensor dataset collected during the TIRAMISU (Thermal InfraRed Anisotropy Measurements in India and Southern eUrope) campaign at the Nawagam research site in Gujarat, India, during the 2023 monsoon season. The objective was to acquire continuous ground-based optical and thermal measurements over a homogeneous rice canopy across different crop growth stages.

The dataset integrates several complementary components. Thermal data were acquired with an Optris longwave infrared camera (8–14 µm) at high temporal resolution, capturing canopy temperature dynamics throughout the diurnal cycle. Optical data were obtained with a Micasense RedEdge-M multispectral sensor, providing imagery in Blue, Green, Red, RedEdge, and Near-Infrared bands with radiometric corrections. An Apogee radiometer supplied reference radiometric temperature. Meteorological measurements included air temperature, humidity, wind speed and direction, and net radiation. Ancillary field measurements comprised Leaf Area Index (LAI), plant height, emissivity sampling, hyperspectral observations, and crop stage information.

The datasets are provided with metadata and processing workflows, including calibration procedures for optical reflectance and thermal radiance. Together, these components form a comprehensive record of canopy–atmosphere interactions over a homogeneous rice field. The datasets can support research on optical and thermal directional anisotropy, canopy radiative transfer, emissivity characterization, and crop biophysical parameter estimation. In addition, they are relevant for applications in vegetation monitoring, agricultural water stress assessment, and surface energy balance studies. By combining optical, thermal, and meteorological observations, the resource is suited for multidisciplinary investigations in remote sensing, agronomy, and environmental sciences.

Specifications Table

**Table:** 

Subject	Earth & Environmental Sciences
Specific subject area	Optical and thermal directional anisotropy over homogeneous crop canopies
Type of data	• Images (thermal infrared, multispectral) • Tables (meteorological variables, canopy structure, emissivity, hyperspectral measurements) • Processed charts/graphs (diurnal cycles, vegetation indices, LAI relationships) • Metadata and calibration workflows
Data collection	Thermal data were collected with an Optris LWIR infrared camera (8–14 µm). Optical data were obtained with a Micasense RedEdge-M multispectral sensor (Blue, Green, Red, RedEdge, NIR) with downwelling light sensor. Reference radiometric temperatures were measured with an Apogee radiometer. Meteorological variables were recorded with a standard weather station. Ancillary canopy parameters were measured with LAI-2200C, plant height gauges, and a hyperspectral spectroradiometer. Emissivity measurements were conducted under controlled laboratory conditions using Bruker's Vertex 80v FTIR spectrometer with Hyperion 3000 microscope operating in 0.6 – 20 μm range. Data were processed with calibration procedures including vignette correction, Fresnel adjustment, and temporal smoothing.
Data source location	Nawagam research site, Gujarat, India Coordinates: 22.796877° N, 72.5748434° E
Data accessibility	**Repository name:** Optical, Thermal Infrared, and Meteorological Dataset from the Thermal InfraRed Anisotropy Measurements in India and Southern eUrope (TIRAMISU) Rice Canopy Experiment **Data identification number: doi.org/10.6096/1028** **Direct URL to data:**https://doi.org/10.6096/1028 **Instructions for access:** Publicly accessible repository; representative subsets provided with metadata and processing scripts.
Related research article	Pinnepalli, C., Roujean, J.-L., Irvine, M., et al. [1]. Measuring and modelling directional effects in the frame of TIRAMISU. *ISPRS Annals, X–3–2024*, 325–330. https://doi.org/10.5194/isprs-annals-X–3–2024–325–2024 Pinnepalli, C., Roujean, J.-L., Irvine, M., et al. [2]. Directional effects in TIR over rice crops: results from the Nawagam site. In *IGARSS 2024 Proceedings*. 10.1109/IGARSS53475.2024.10641023

## 1 Value of the Data

• Provides a unique multi-sensor dataset (optical, thermal infrared, meteorological, and ancillary field data) collected over a homogeneous rice canopy.
• Enables reuse for studying optical and thermal directional anisotropy, radiative transfer, and emissivity characterization.
• Includes ancillary LAI, plant height, hyperspectral reflectance, and laboratory emissivity data, useful for crop parameter validation.
• Supplied with metadata and calibration workflows, ensuring reproducibility and adaptability by other researchers.
• Supports applications in vegetation monitoring, water stress assessment, and surface energy balance across remote sensing, agronomy, and environmental sciences.

This dataset represents irrigated paddy rice under semi-arid monsoon conditions at a single managed research site. Users should be cautious when transferring empirical relationships to other rice systems, but the dataset remains broadly useful for directional anisotropy, radiative transfer, and retrieval method testing because the geometry, metadata, and processing workflows are transferable.

## 2 Background

The TRISHNA (Thermal infraRed Imaging Satellite for High-resolution Natural resource Assessment) mission, jointly developed by CNES and ISRO, is designed to provide global observations of land surface temperature (LST) and evapotranspiration with high spatial (57–90 m) and temporal (3–5 days) resolution. Achieving these objectives requires precise radiometric calibration and correction of directional anisotropy effects in thermal infrared (TIR) measurements, particularly around the hotspot geometry where sun–sensor alignment can bias LST retrievals by several kelvins. Such directional effects, if uncorrected, propagate into evapotranspiration estimates and water stress monitoring, the primary deliverables of TRISHNA.

To address this challenge, the TIRAMISU (Thermal InfraRed Anisotropy Measurements in India and Southern eUrope) experiment was initiated to acquire high-frequency multi-angular datasets of optical and thermal measurements over vegetated canopies. These datasets enable the characterization of TIR anisotropy and support the validation of physical (e.g., SCOPE [3], DART [4]) and parametric BRDF models used to normalize directional effects in satellite observations.

The Nawagam site in Gujarat, India, was selected for such measurements due to its homogeneous paddy rice fields and established research infrastructure. The site is located at the Main Rice Research Station of Anand Agricultural University (**22.796877° N, 72.5748434° E**). This experimental farm falls under the semi-arid (Zone-III) agro-climatic region of Gujarat with annual temperatures from 21 °C to 43 °C.

The site is equipped with a flux tower for micrometeorological observations and additional sensors for optical and thermal measurements. A MicaSense Red Edge multispectral camera and an Optris PI640 longwave infrared camera (8–14 µm) were mounted on a motorized mast to collect multi-angular directional datasets over the rice crop during the 2023 growing season (July–October). Supporting instruments included Apogee thermal radiometers, soil temperature and moisture probes, and meteorological sensors (wind, air temperature, air humidity).

This experimental setup was jointly supported by Anand Agricultural University, the Space Applications Centre (ISRO), and CNES as part of the Indo-French collaboration on thermal infrared measurements. Together with ancillary field campaigns (leaf area index, canopy height, emissivity, and hyperspectral reflectance), these datasets provide a comprehensive basis for analyzing directional anisotropy in paddy rice canopies under semi-arid monsoon conditions.

## 3 Data Description

The mentioned repository has two versions of datasets to download: Sampled dataset for the testing and quick access (/TRISHNA_TIRAMISU_PUBLICATION_Nawagam_SampledDataset/). Another version with complete dataset along with sampled dataset for references (/TRISHNA_TIRAMISU_PUBLICATION_Nawagam_CompleteDataset). Each dataset version is organized into the following main directories, each containing raw measurements, processed outputs, and supporting metadata.

1. **/Meteo_Data/**
  • **meteo_raw.xlsx** — High-frequency meteorological data (1-minute resolution).
    ○ Variables: air temperature (°C), relative humidity (%), wind speed (m·s^−1^), wind direction (°), net radiation (W·m⁻²).
  • **flux_tower.xlsx** — Half-hourly averaged flux data from the eddy covariance tower.
    ○ Variables: sensible heat flux (H, W·m⁻²), latent heat flux (LE, W·m⁻²), CO₂ flux (µmol·m⁻²·s^−1^), H₂O flux (mmol·m⁻²·s^−1^), air pressure (kPa), vapor density (mmol·m⁻³), and friction velocity (u*).
  • **Datalogger_data.ods** — Raw data stream exported directly from the Campbell CR3000 datalogger, containing continuous records from tower-mounted sensors (radiation, air temperature, humidity, wind speed/direction, soil probes). This file preserves the unprocessed logger outputs before quality control and averaging.
    ○ Variables: logger timestamps, sensor voltages, raw micrometeorological readings.
2. **/Ancillary_Measurements/**
  Contains additional field and laboratory measurements used to complement the thermal and optical remote sensing data.
  • canopy_measurements.xlsx
    ○ Field observations of vegetation and water status.
    ○ Variables: leaf area index (LAI, m²·m⁻²), plant height (cm), water depth (cm), sampling date.
  • /Emissivity_Measurements/
    ○ Subfolders organized by campaign date (e.g., 01092023, 10082023).
    ○ Each folder contains OpenDocument spreadsheets (.ods) with spectral measurements of leaf reflectance and transmittance, separated into *Top of Leaf* and *Bottom of Leaf* for multiple samples.
    ○ Variables: spectral reflectance (%) and transmittance (%) across thermal and optical wavelengths.
  • /HyperSpectral_Measurements/
    ○ Hyperspectral reflectance campaigns, organized in dated subfolders (e.g., 01_09_2023).
    ○ Each dated folder includes three subcategories:
      ▪ reference/ — calibration targets
      ▪ vegetationleaf/ — canopy/leaf samples
      ▪ water/ — water surface samples
    ○ Files are stored as .sed (spectral data) files.
    ○ Variables: reflectance spectra from 350–2500 nm (1 nm resolution).
  • /Gas_Concentrations_LI7810/
    ○ High-frequency trace gas concentration data collected with the LI-COR LI-7810.
    ○ Files: Excel spreadsheets named by date (e.g., Nawagam_LI7810_01092023.xlsx).
    ○ Variables: CO₂ (ppm), CH₄ (ppb), H₂O (ppm), cavity pressure (kPa), cavity temperature (°C), diagnostic residuals, laser power, quality flags.
3. **/Scripts_sampled_data/**
  This folder contains sample datasets and processing code for reproducibility.
  • /Thermal_CSV_Files/ — Example raw Optris thermal CSVs (per-frame).
  • /Thermal_Output/ — Sample processed PNGs after undistortion.
  • /Micasense_TIFF_Files/ — Example MicaSense RedEdge TIFFs (Blue, Green, Red, RedEdge, NIR).
  • /RGB_Output/ — Example RGB composites generated from the TIFFs.
  • Thermal_CSV_to_Image.py — Python script to process Optris CSVs into images.
  • Imageprocessing_RedEdge_Nawagam.zip — Full Git-based MicaSense processing environment (including the Nawagam_Micasense_ImageProcessing.ipynb notebook).
  • README_Nawagam.md — Instructions for environment setup and script execution.
4. README_Full_Nawagam.md

Provides dataset overview, folder organization, variable definitions, software requirements, and usage notes.

## 4 Experimental Design, Materials and Methods

### 4.1 Study site

Measurements were conducted at the **Main Rice Research Center, Anand Agricultural University (AAU), Nawagam, Gujarat, India** (22.796877° N, 72.5748434° E; altitude ∼25 m). The site represents a homogeneous irrigated paddy rice field located in the semi-arid Zone-III agro-climatic region of Gujarat. The local climate during the rice-growing season is marked by moderate air temperatures (15–33 °C) and large fluctuations in relative humidity (30–100 %) associated with irrigation and monsoon rainfall. Wind speeds are typically 5–12 m s^−1^ during the monsoon months (May–August) and decrease to 2–5 m s^−1^ in summer and autumn. Annual rainfall varies between 250 and 900 mm, with most precipitation occurring during the monsoon period.

**Fig. 1:**
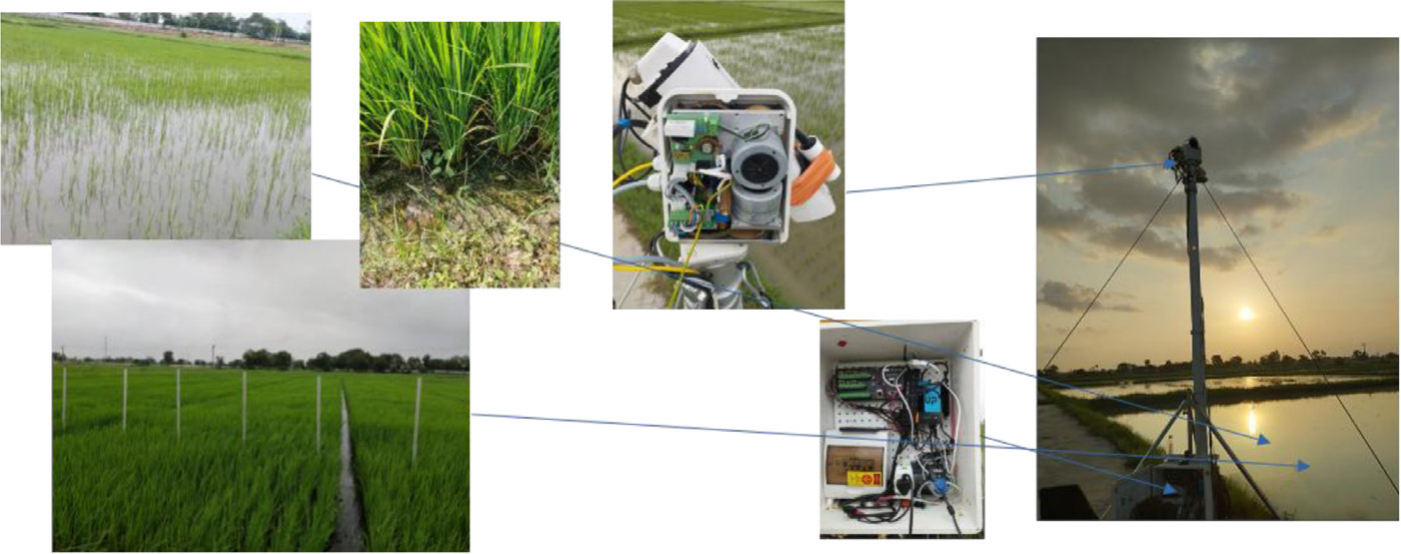
Location of the Nawagam research site (AAU, Gujarat, India) with experimental layout (thermal and multispectral sensors).

The site is equipped with an **eddy covariance flux tower** and supporting meteorological sensors, making it suitable for continuous monitoring of canopy–atmosphere interactions. During the 2023 rice growing season (July–October), combined **thermal, multispectral, meteorological, and ancillary field measurements** were collected to document canopy conditions and directional anisotropy. Fig. 1 illustrates the overall instrumentation and layout of the site during the observing period.

### 4.2 Instrumentation

A coordinated multi-sensor setup was deployed to collect thermal, multispectral, meteorological, and ancillary field measurements.

**Thermal camera (Optris PI640):** Longwave infrared imager (8–14 µm), 640 × 480 pixels, field of view 60° × 45° Connected to a laptop running PIX Connect software for real-time acquisition. Mounted on a Videotec PTH-900 pan–tilt head with ∼40 programmable azimuth/zenith geometries. This setup enables systematic directional sampling required for studying anisotropy in the TIR domain, as discussed in [5,6].

**Multispectral camera (MicaSense RedEdge-M):** Captures 5 bands: Blue (475 nm), Green (560 nm), Red (628 nm), RedEdge (717 nm), NIR (842 nm). Resolution 1280 × 960 pixels, field of view 47.2° × 35.4° Installed on the same mast as the Optris for synchronized acquisitions, allowing comparison between optical and thermal angular responses.

**Thermal radiometers (Apogee SI-431, SI-131/111):** Narrow field-of-view sensors mounted at fixed orientations for independent canopy temperature validation.

**Flux tower instruments:** LI-COR LI-7500 CO₂/H₂O analyzer, Gill 3D sonic anemometer, Apogee net radiometers, soil moisture probe (ML2X ThetaProbe), soil thermocouples (T107), all logged via a Campbell CR3000 datalogger.

**Ancillary instruments:** LAI-2200C for canopy structure, SPAD-502 Plus for chlorophyll, Spectral Evolution (350–2500 nm, 1 nm) for hyperspectral reflectance, laboratory spectrometer for emissivity (leaf reflectance/transmittance), LI-COR LI-7810 gas analyzer for CO₂, CH₄, H₂O (1 Hz).

**System integration:** Power via 220 V line with backup storage; remote access via Anydesk; hourly webcam monitoring.

**Thermal infrared measurements (Optris PI640):**

**Figure 2:**
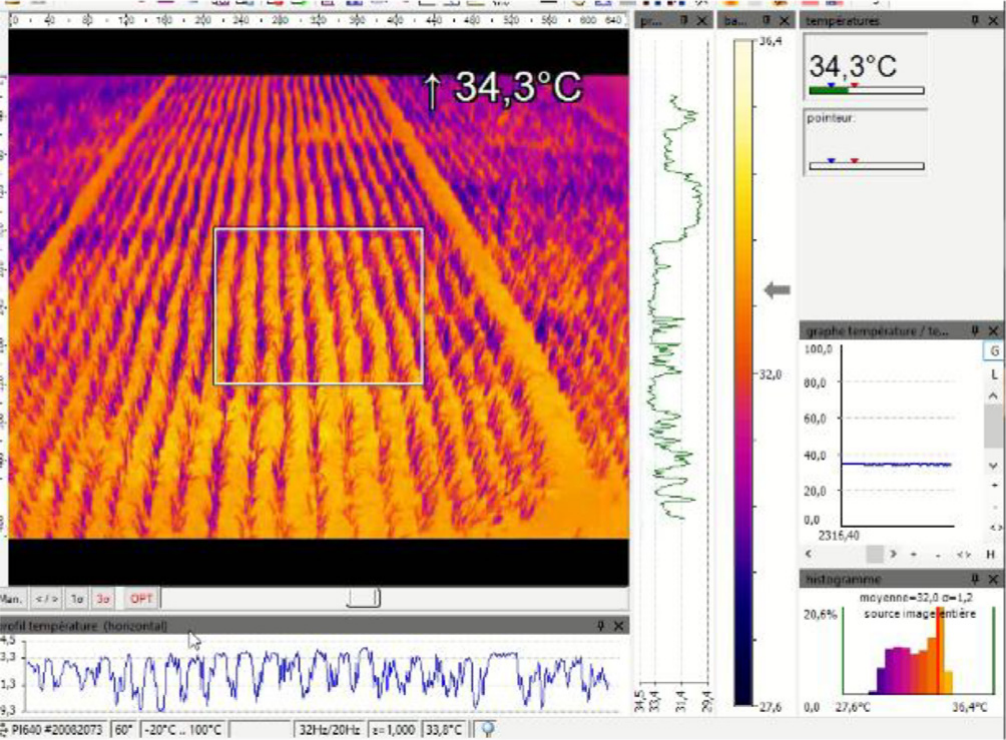
PIX Connect software visualization of a thermal image acquired with the Optris PI640.
• **Variables measured**: Brightness temperature per pixel (°C), timestamp, acquisition geometry.

• **Protocol**: Continuous imaging 09:00–17:00 IST (July–October 2023) across crop stages. Images captured at each programmed view angle to maximize directional coverage for BRDF-like analysis in the thermal domain [7].
• **Processing:**
  ○ Exported as .csv with per-pixel brightness values.
  ○ Reshaped into 2D arrays (640 × 480).
  ○ Lens distortion corrected using **OpenCV** with calibrated coefficients (k1 = –0.42230, k2 = 0.52240, k3 = –0.64157, p1 = 0.00003, p2 = 0.00185).
  ○ Outputs exported as .png thermal images for visualization. Fig. 2 presents the interface layout of the PIX Connect software used for image display, video playback, and live monitoring of the Optris camera.

**Optical multispectral measurements (MicaSense RedEdge-M):**

**Fig. 3:**
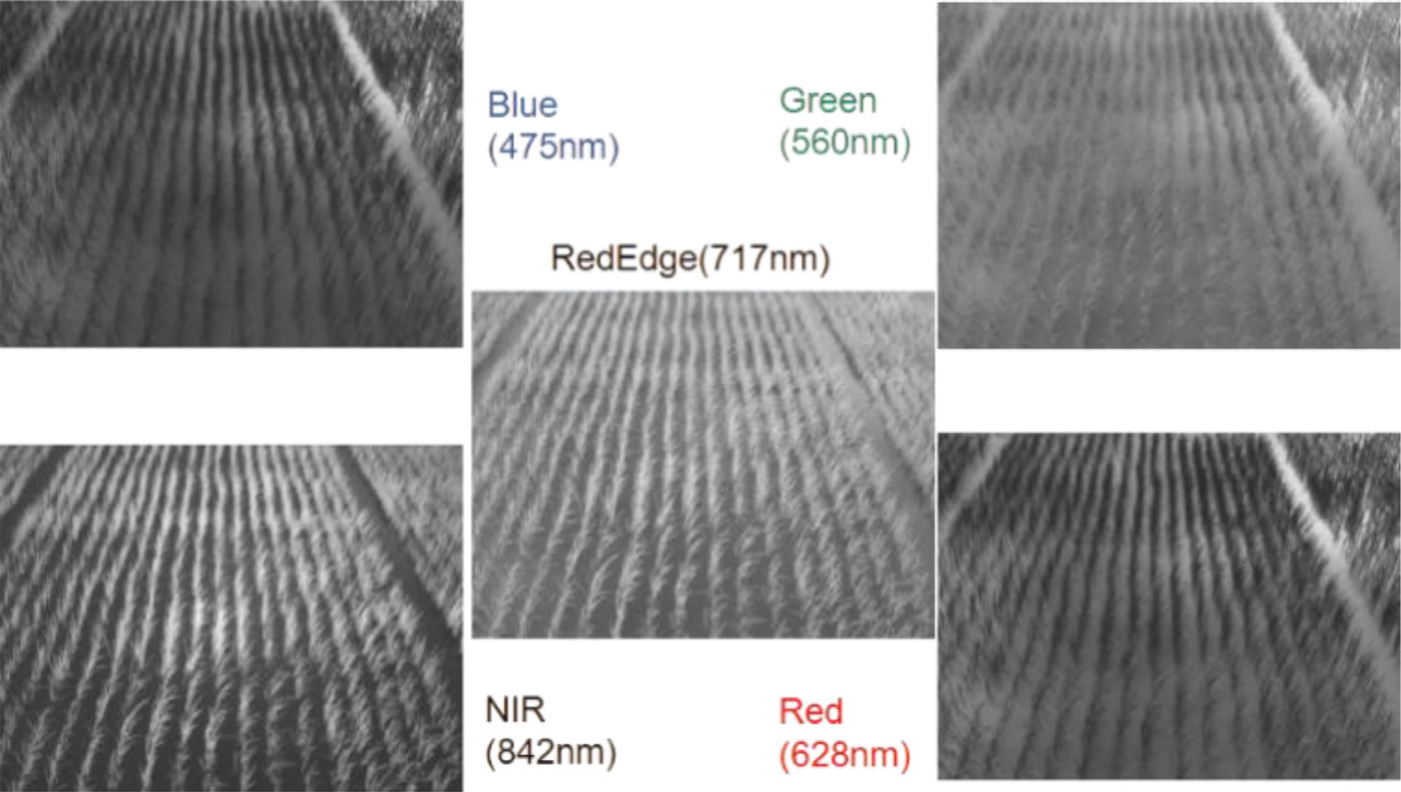
MicaSense RedEdge-M band images (Blue 475 nm, Green 560 nm, Red 628 nm, Red Edge 717 nm, NIR 842 nm) visualized.
• **Processing:**
  ○ Radiometric corrections (exposure, vignetting, gain).
  ○ Band alignment applied with NIR as reference using homography warp.
  ○ RGB composites generated from Red, Green, Blue with percentile normalization (0.5–99.5%).
• **Variables measured**: Spectral radiance values, RGB composites.

• **Protocol**: Captures 5 monoband TIFFs simultaneously (Blue, Green, Red, RedEdge, NIR) during the same viewing geometry as Optris thermal acquisitions. Fig. 3 shows sample raw images from the individual spectral bands.

### 4.3 Temporal alignment and synchronization

Thermal (Optris PI640) and multispectral (MicaSense RedEdge-M) acquisitions were synchronized by mounting both sensors on the same mast and applying the same programmed viewing geometries. For each viewing sequence, optical and thermal files share a common acquisition time reference and can be matched using the metadata fields provided in the repository. Meteorological measurements were recorded continuously at 1-min resolution and hourly resolution for some variables, enabling direct temporal matching to each image timestamp.

Ancillary field observations (LAI, plant height, water depth, phenology notes) were collected on predefined campaign days and linked to imaging observations acquired on the same date.

### 4.4 Meteorological and flux measurements

• **Meteorology**: 1-min resolution air temperature, humidity, wind speed/direction, net radiation. Installation heights above ground level (AGL) for the meteorological and eddy-covariance sensors are listed in Table 1, referenced to the soil surface at the tower base.

**Table 1:** Sensor installation heights.

Variable	Height (m AGL)
Air temperature & relative humidity	2.5
Wind speed/direction	5.0
CO₂/H₂O concentrations	5.0
Net radiation	2.0
Tower height	10.0
• **Eddy covariance**: 30-min averages of sensible heat flux (H), latent heat flux (LE), CO₂ and H₂O fluxes, vapor density, air pressure, and friction velocity (u*). These data provide reference energy balance variables supporting the optical–thermal observations, as in previous multi-sensor canopy studies [1].
• All tower-mounted sensors were logged via a Campbell CR3000 datalogger, whose raw outputs are preserved in the file Datalogger_data.ods for reproducibility.

### 4.5 Ancillary measurements

• **Canopy structure**: LAI with LAI-2200C; canopy height and water depth recorded manually.
  LAI was measured at 10 locations per date distributed across the plot. Plot-level LAI in *canopy_measurements.xlsx* is reported as mean across locations.
• **Emissivity**: Leaf reflectance and transmittance spectra from the top and bottom surfaces were measured under controlled laboratory conditions. Samples were grouped into two categories, with preserved leaves frozen to maintain moisture content and minimize environmental interactions, while non-preserved leaves were transported without protection from ambient conditions. Preservation was implemented to reduce potential alterations in leaf properties during the 90 min transport interval from the field site to the laboratory.
• Two emissivity datasets are provided: one derived from reflectance only (ε = 1 − ρ) and another including both reflectance and transmittance (ε = 1 − ρ − τ). Accounting for transmittance yields more realistic values for partially translucent leaves, whereas ignoring it tends to overestimate emissivity [8].
• **Hyperspectral**: Spectral Evolution spectra (350–2500 nm, 1 nm) over vegetation, water, and reference targets.
• **Trace gases**: LI-7810 analyzer at 1 Hz, recording CO₂, CH₄, H₂O concentrations, plus pressure, temperature, and diagnostics.

### 4.6 Scripts and reproducibility

**Fig. 4:**
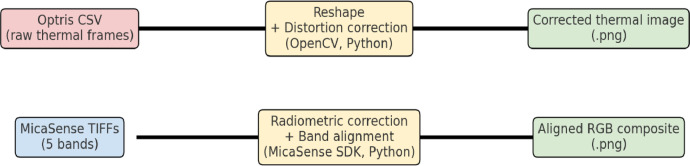
Workflow from raw acquisition to processed outputs: Optris CSV reshaped and distortion-corrected; and MicaSense TIFFs aligned into RGB composites.

• **Thermal_CSV_to_Image.py**: Python script for Optris data reshaping, distortion correction, and PNG export.
• **Imageprocessing_RedEdge_Nawagam.zip**: Repository for MicaSense processing for band alignment and RGB generation.
• **Dependencies**: Python ≥ 3.9 with NumPy, OpenCV-python, matplotlib, scipy, scikit-image, imageio, exifread, pytz.
• The inclusion of scripts ensures transparency and reproducibility of the processing workflow, complementing prior BRDF/TIR methods development in anisotropy studies [2]. Fig. 4 presents a flowchart of the basic image processing workflow for both Optris and MicaSense data.

The combined deployment of mast-mounted thermal and multispectral sensors, flux tower measurements, and targeted field campaigns provided synchronized, multi-modal observations of irrigated rice under semi-arid monsoon conditions. Acquisition protocols ensured temporal coverage across growth stages and angular diversity for anisotropy analysis. With raw measurements, processed outputs, and open-source scripts included, the dataset supports transparency, reproducibility, and broad reuse across remote sensing, agronomy, and environmental sciences. Table 2 summarises all the component discussed in this section.

**Table 2:** Dataset components and typical applications.

Data type	Contents (examples)	Typical applications
Thermal infrared images	Time series of images	TIR directional anisotropy studies, LST/emissivity sensitivity
Optical images	Time series of images	Optical BRDF/anisotropy, vegetation indices, biophysical retrieval
Meteorology	Air T/RH, wind, net radiation, rainfall	Context for canopy–atmosphere exchange, forcing for energy balance models, Quality Check screening
Eddy covariance / fluxes	LI-7500 + sonic derived flux products	Surface energy balance and turbulent exchange
Ancillary canopy measurements	LAI, plant height, water depth, phenology notes	Validation/constraint for retrieval models, linking structure to directional signals, structural measurements for RT models
Emissivity measurements	emissivity estimates	Emissivity parameterization, LST retrieval uncertainty analysis
Spectral measurements	Hyperspectral reflectance	reflectance studies, spectral index development, cross-sensor harmonization
Metadata & scripts	geometry, timestamps, calibration notes, processing code	Reproducibility, standardized fusion,

## Limitations

The dataset is extensive, covering thermal, multispectral, meteorological, and ancillary measurements throughout the rice growth cycle. Some practical challenges occurred during field campaigns, including occasional power fluctuations and mast motor malfunctions; these were resolved by redundant systems and fixing the camera orientation to ensure continuity. Optical composites (MicaSense) are radiometrically corrected and normalized, but not fully calibrated with panel/DLS data for the same instant, making them most suitable for visualization and relative analysis. Hyperspectral, emissivity, and gas concentration measurements were collected at specific crop stages or campaigns, providing representative snapshots rather than continuous series. Despite these factors, the dataset provides rare, synchronized, multi-sensor observations over rice canopies, together with processing scripts to ensure reproducibility.

The data covers a single growing season (2023 monsoon; July–October) and therefore is not designed for interannual variability analyses or long-term climate trend assessments. Its main strength is dense, synchronized multi-sensor sampling across crop growth stages within one season, supporting method development, directional normalization studies, and process-level evaluation.

## Ethics Statement

This work does not involve human subjects, animal experiments, or social media data.

## CRediT Author Statement

**Chandrika Pinnepalli:** Data curation; Methodology; Validation; Visualization; Writing – original draft; Funding acquisition. **Jean-Louis Roujean:** Conceptualization; Supervision; Methodology; Writing – review & editing, Funding acquisition. **Mark Irvine:** Methodology; Resources. **Marc Oliver-Soulayrol:** Data curation; Investigation; Validation; Visualization; Resources. **Rahul Nigam:** Investigation; Data curation; Resources. **Ayan Das:** Investigation; Data curation; Resources. **Bimal Bhattacharya:** Investigation; Data curation; Resources. **Rucha Dave:** Investigation; Data curation; Resources.

## Data Availability

Service de données de l'Observatoire Midi-PyrénéesOptical and Thermal Infrared Image Dataset from the Thermal InfraRed Anisotropy Measurements in India and Southern eUrope (TIRAMISU) Rice Canopy Experiment (Original data).
